# Rapid initiation of antiretroviral therapy in Turkey: a modeling study

**DOI:** 10.3389/fpubh.2024.1224449

**Published:** 2024-01-26

**Authors:** Emine Yaylali, Zikriye Melisa Erdogan, Fethi Calisir, Husnu Pullukcu, Figen Yildirim, Asuman Inan, Ozlem Altuntas Aydin, Suda Tekin, Meliha Cagla Sonmezer, Toros Sahin, Tahsin Gokcem Ozcagli, Berna Ozelgun

**Affiliations:** ^1^Department of Industrial Engineering, Istanbul Technical University, Istanbul, Türkiye; ^2^Infectious Diseases, Ege University, Izmir, Türkiye; ^3^Infectious Diseases and Clinical Microbiology, Akdeniz Yasam Hospital, Antalya, Türkiye; ^4^Infectious Diseases and Clinical Microbiology, Haydarpasa-Numune Training and Research Hospital, Istanbul, Türkiye; ^5^Infectious Diseases and Clinical Microbiology, University of Health Sciences, Istanbul, Türkiye; ^6^Infectious Diseases and Clinical Microbiology, Koc University, Istanbul, Türkiye; ^7^Infectious Diseases and Clinical Microbiology, Hacettepe University, Ankara, Türkiye; ^8^Gilead Sciences, Istanbul, Türkiye

**Keywords:** HIV infections, HIV care-continuum, mathematical modeling, rapid antiretroviral treatment, infectious disease modeling

## Abstract

**Background:**

To effectively control the HIV epidemic and meet global targets, policymakers recommend the rapid initiation of antiretroviral therapy (ART). Our study aims to investigate the effect of rapid ART programs on individuals diagnosed with HIV, considering varying coverage and initiation days after diagnosis, and compare it to standard-of-care ART treatment in Turkey.

**Methods:**

We used a dynamic compartmental model to simulate the dynamics of HIV infection in Turkey. Rapid treatment, defined as initiation of ART within 7 days of diagnosis, was contrasted with standard-of-care treatment, which starts within 30 days of diagnosis. This study considered three coverage levels (10%, 50%, and 90%) and two rapid periods (7 and 14 days after diagnosis), comparing them to standard-of-care treatment in evaluating the number of HIV infections between 2020 and 2030.

**Results:**

Annual HIV incidence and prevalence for a 10-year period were obtained from model projections. In the absence of a rapid ART program, the model projected approximately 444,000 new HIV cases while the number of cases were reduced to 345,000 (22% reduction) with 90% of diagnosed cases included in the rapid ART program. Similarly, 10% and 50% rapid ART coverage has resulted in 3% and 13% reduction in HIV prevalence over a 10-year period.

**Conclusion:**

Rapid ART demonstrates the potential to mitigate the increasing HIV incidence in Turkey by reducing the number of infections. The benefit of the rapid ART program could be substantial when the coverage of the program reaches above a certain percentage of diagnosed population.

## 1 Introduction

As of the end of 2019, among the estimated 38 million people living with HIV globally, 67% of them had access to treatment in the world (1). With the help of global prevention and intervention efforts, the proportion of people living with HIV (PLWH) on antiretroviral therapy (ART), which is known as treatment coverage, is increasing. Consequently, HIV incidence has been decreasing or stabilizing in many countries. New HIV prevention targets aim to achieve 95% of PLWH know their HIV status, 95% of those aware of their HIV positive status accessing treatment, and 95% of people on treatment having suppressed viral loads by 2030. However, since 2020, these targets have been missed (1), with 1.5 million new HIV infections in that year. There is still much more effort to make to meet the 2030 targets.

Following the “treat all” strategy, Rapid ART Program for Individuals with an HIV Diagnosis (RAPID) is recognized as one of the key recommendations in the fight against the HIV infection. Ideally, rapid ART involves initiating treatment in the first 24 h of diagnosis, it is defined as the initiation of ART within 7 days of diagnosis (2), while some studies considered initiation within < 14 days after diagnosis (3, 4). This strategy has been recommended by several guidelines, including the World Health Organization (2), the U.S. Department of Health and Human Services (5), and the International Antiviral Society (4).

The initiation of ART as early as HIV diagnosis has been an important public health strategy for HIV prevention due to two main reasons. First, rapid ART initiation enables viral load suppression (VLS) much faster (6, 7) and resulting viral suppression may prevent HIV transmission since treatment prevents up to 93–96% of infections in serodiscordant couples (i.e., treatment as prevention- TasP) (8, 9). Second, early initiation of treatment may decrease mortality and morbidity and improve the health-related quality of life (HRQoL) among people living with HIV (PLWH) (3).

Rapid ART initiation has been evaluated by randomized controlled trials and assessed by observational studies. These randomized trials [RapIt (10), START-ART (11), same day ART in Haiti (12), and CASCADE (13)] and observational studies [RAPID in San Francisco (14), TRRT in Miami (15) and CCSI in New Orleans (16)] have determined that patients in the rapid ART arm exhibit higher rates of linkage to care, retention in care and viral suppression. Similar results have been found in a systematic review on rapid ART initiation, reporting that rapid ART improves patient outcomes compared to standard-of-care treatment (3). Many countries globally have embraced rapid ART implementation in accordance with the latest guidelines. Data presented in 2023 reveal that 99 countries, corresponding to 81% of 122 reporting countries, have adopted WHO's recommendation to offer rapid ART, providing ART on the same day as HIV diagnosis. This fact represents 46% increase from 68 countries reported in 2020 worldwide (17).

Since the publication of the national guideline in 2013, ART has been the recommended as a standard treatment for all HIV-positive individuals, regardless of their CD4 cell count in Turkey. Moreover, initiation of treatment generally takes 2–4 weeks (18). However, new HIV-positive cases have been steadily increasing in the last decade in the country with nearly 60% of cases diagnosed in the last 5 years. The spread of HIV in Turkey is obscured by various contributing factors, including a lack of knowledge and awareness about the disease, being one of the most popular tourism destinations increasing the risk of exposure, high population mobility, an increasing number of unregistered sex workers, lack of prevention measures focusing on key risk groups, insufficient number of Voluntary Counseling and Testing (VCT) centers, persistent stigma, and discrimination against PLWH (19). Turkish Ministry of Health (MoH) conducted the National HIV/AIDS Control Program to enhance public health and create the roadmap for HIV in Turkey in 2019. One of the important goals in this program is to decrease the number of new HIV cases and deaths due to HIV. Moreover, it was stated that the main approach to decrease HIV transmission could be achieved by providing early diagnosis, access to care, and retention in care for PLWH, leading to viral suppression (20). Thus, we can conclude the programs that an increase in viral suppression and a decrease in transmission risk would be valuable in achieving the national goal of reduced number of new HIV positive cases. The previous studies show that the current situation in the country ranges between 48 and 50% regarding HIV diagnosis, 75.3 and 88% for ART coverage, and 85 and 87% for VLS rates (21, 22). Rapid initiation of ART would play a crucial role in improving the current continuum of care, and it should be analyzed for further consideration as it can be beneficial in reducing the total number of cases. Our motivation is to investigate the effectiveness of introducing a rapid ART initiation program in both mitigating the increasing trend of HIV and improving the current HIV prevention strategies in Turkey.

To the best of our knowledge, there are no published studies regarding rapid ART initiation in Turkey. In this study, our objective is to evaluate the differences in effects between implementing a rapid initiation of the ART program and standard care over a 10-year period in Turkey. Thus, the goal is to assess the benefit of rapid ART programs in Turkey and quantify these benefits by determining the number of prevented infections, if exists.

## 2 Materials and methods

### 2.1 Model structure

We adopted a previously developed HIV transmission and progression model of Turkey for rapid ART initiation (23). The deterministic dynamic compartmental model was tailored for a portion of diagnosed PLWH to participate in the onset of rapid ART program while the remaining diagnosed PLWH proceed with the standard treatment. The model was developed in MATLAB environment (24) and formulated using ordinary differential equations (ODEs) with details of the system as given in the Appendix.

The model population was stratified based on the disease status (HIV negative and HIV positive) and transmission risk [men who have sex with men (MSM), people who inject drugs (PWID), and heterosexuals (HET)]. PLWH in the model is further divided into subpopulations based on disease stages and continuum of care. As a result, aside from susceptible (HIV negative) and death due to HIV compartments, PLWH is divided into the following compartments: CD4 count>200 cells/mm^3^ and undiagnosed, CD4 count < 200 cells/mm^3^ and undiagnosed, CD4 count>200 cells/mm^3^ diagnosed but not on ART, CD4 count < 200 cells/mm^3^ diagnosed but not on ART, CD4 count>200 cells/mm^3^ diagnosed, on ART but no viral load suppression (VLS), CD4 count < 200 cells/mm^3^ diagnosed, on ART but not VLS, CD4 count>200 cells/mm^3^ VLS, and CD4 count < 200 cells/mm^3^ VLS. Model formulation is presented in the model formulation section of Appendix and Supplementary Table A2.

**Figure 1:**
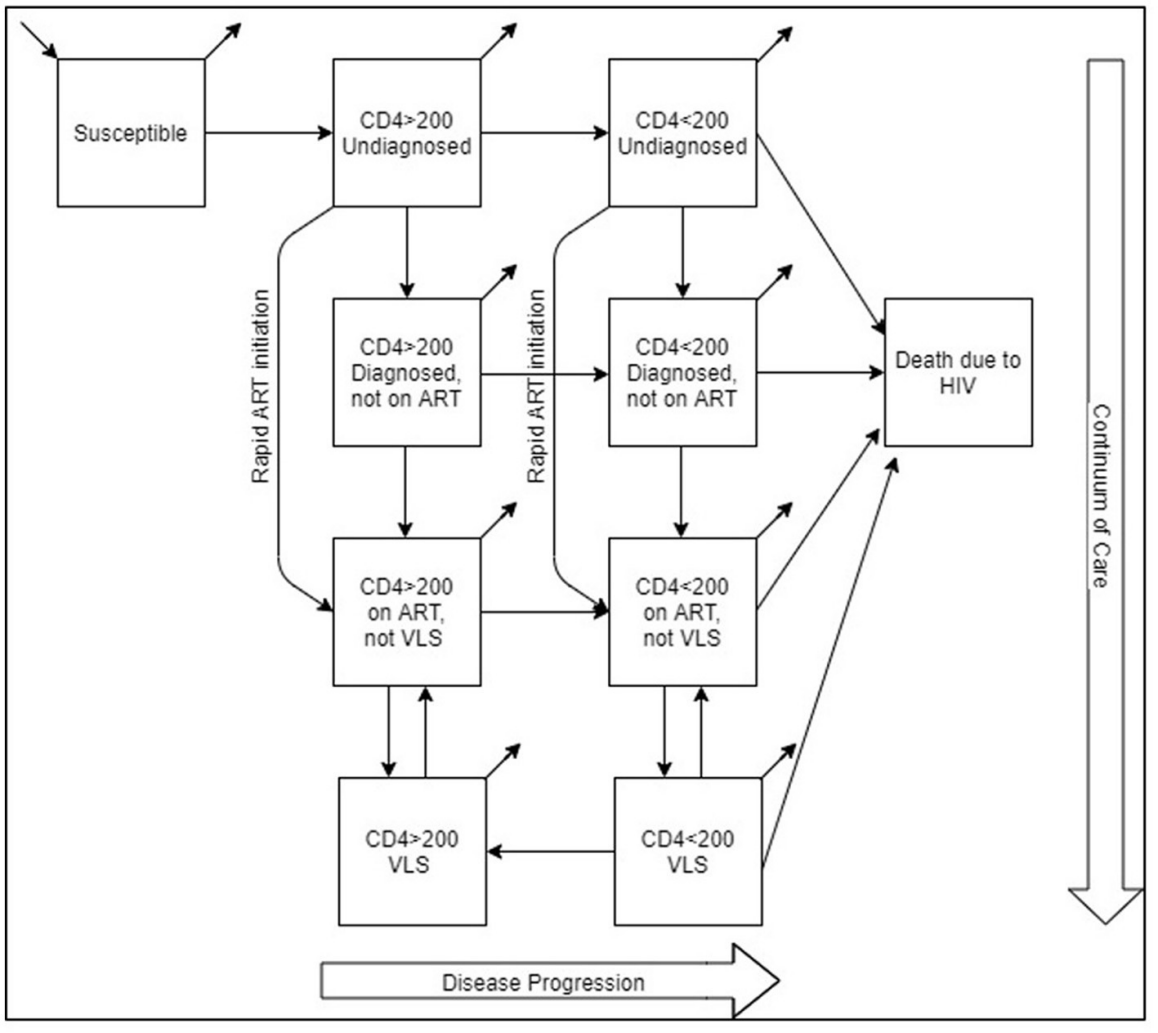
Model flow diagram including rapid ART initiation.

Rapid ART initiation is integrated with the model via a flow between two sets of compartments. Individuals diagnosed and initiated ART in the “rapid” program move directly from undiagnosed compartments (CD4 count>200 cells/mm^3^ or CD4 count < 200 cells/mm^3^) to the “on ART not VLS” compartments, bypassing diagnosed but not on ART compartments, which represents the standard-of-care ART initiation route (Figure 1). Since there is currently no rapid ART initiation in Turkey, we generated the “rapid” flow rates by three levels of coverage and the length of the rapid period.

Rapid ART leads to faster VLS compared to delayed treatment. In the model schematic, this is accomplished by bypassing the “Diagnosed not on ART” compartment, allowing individuals to move faster to the “on ART not VLS” compartment. The parameters for achieving VLS or drop out of VLS remain consistent across scenarios, but the time taken to reach VLS compartment decreases due to earlier entry into the on ART compartments. By achieving VLS faster, patients become less infectious, effectively curbing the spread of HIV within the population in the model. Thus, rapid ART serves as a critical component of HIV prevention efforts.

The model was validated against the number of confirmed cases reported by the Ministry of Health and considered a time horizon of 2010–2019 as its fitting (calibration) period. The prediction period extended 2020 and beyond. The primary model predictions focused on outcomes related to HIV incidence and prevalence between 2020 and 2030 and the effect on continuum of care during the same period.

### 2.2 Model parameters

The model is populated with demographic, epidemiological, behavioral, and clinical data. Input parameters included population size by transmission risk group, continuum of care, mortality rate (HIV-related and non-HIV related), HIV progression among disease stages, and reduction in HIV transmission after diagnosis and treatment (Supplementary Table A1). Parameters related to prevalence rates by transmission group, treatment and VLS rates, HIV-related mortality parameters, birth and natural death rates, and related population parameters are among the country-specific parameters. Due to the scarcity of behavioral parameters based on each HIV transmission group, we calibrated force of infection parameter. Parameters are estimated from several sources, such as medical literature, Turkish Statistical Institute, and three large patient cohorts. These cohorts reported around 8,000 patients, which was ~50% of all reported cases in Turkey between 01/2010 and 12/2019 (23). Data analysis and parameter estimation were conducted using MS Excel. Data extracted from cohort databases were anonymized before access and analysis; thus, no informed consent and/or a consent waiver was obtained during the study. Ethics approval was not required for this modeling study.

Parameters related to rapid ART initiation include the rate at which PLWH diagnosed and initiated in the rapid ART program based on the disease stage and the length of rapid period. These parameters are based on rapid ART initiation scenarios developed due to lack of real-life data on the rapid initiation in Turkey. Once initiated, both rapid ART program and standard ART program have assumed to have same treatment efficacy in reducing the disease transmission and dropping out rates of ART.

### 2.3 Model calibration

To calibrate the model, we generated ranges for input parameters that could not be estimated from datasets and literature and applied the ranges against the reported number of cases based on the disease stage reported by MoH between 2010 and 2019. Details of the calibration procedure are provided in the Appendix.

### 2.4 Rapid ART initiation scenarios

To include rapid ART initiation, we considered six scenarios, which are hypothetical due to lack of real-life data on rapid ART initiation in Turkey (Table 1). In the scenarios, we assumed that different proportions of PLWH diagnosed and associated with care receive the rapid ART while the rest of diagnosed population initiate ART in the standard way. This was considered as the rapid ART coverage. Three coverage levels were 10%, 50%, and 90% of diagnosed PLWH being included in the rapid ART program. These scenarios compared to the base case scenario where no rapid ART program exists and diagnosed PLWH initiates ART ~30 days after the diagnosis. Duration of rapid period was selected as 7 days, representing the relevant guidelines. We also evaluated a rapid ART period of 14 days to investigate the effect of the change in ART initiation time. We assumed that all diagnosed patients are eligible for the rapid ART initiation, the patients offered the rapid treatment do not refuse it, and the coverage of the rapid ART program remains constant over the time horizon. We collected annual HIV incidence, cumulative HIV cases, the number of HIV cases prevented, and the percent reduction in HIV incidence with the rapid ART intervention between 2020 and 2030. We also reported continuum of care for the base case and rapid ART scenarios.

**Table 1:** Scenarios defined under different coverage levels and rapid ART periods.

**Scenarios**	**Coverage level**	**Rapid ART period**
Scenario 1	10%	7 days
Scenario 2	10%	14 days
Scenario 3	50%	7 days
Scenario 4	50%	14 days
Scenario 5	90%	7 days
Scenario 6	90%	14 days

We also applied sensitivity analysis, and further details are explained in Sensitivity Analysis section in the Appendix; then, results of two sensitivity analyses are explained in Supplementary Figures A1, A2.

## 3 Results

The standard-of-care ART, considered as the base case scenario, represents the current healthcare system practice, where HIV-infected persons can access ART 30 days after their diagnosis. Three rapid ART scenarios were defined based on the proportion of patients benefiting from rapid ART intervention, such as 10%, 50% and 90%.

**Figure 2:**
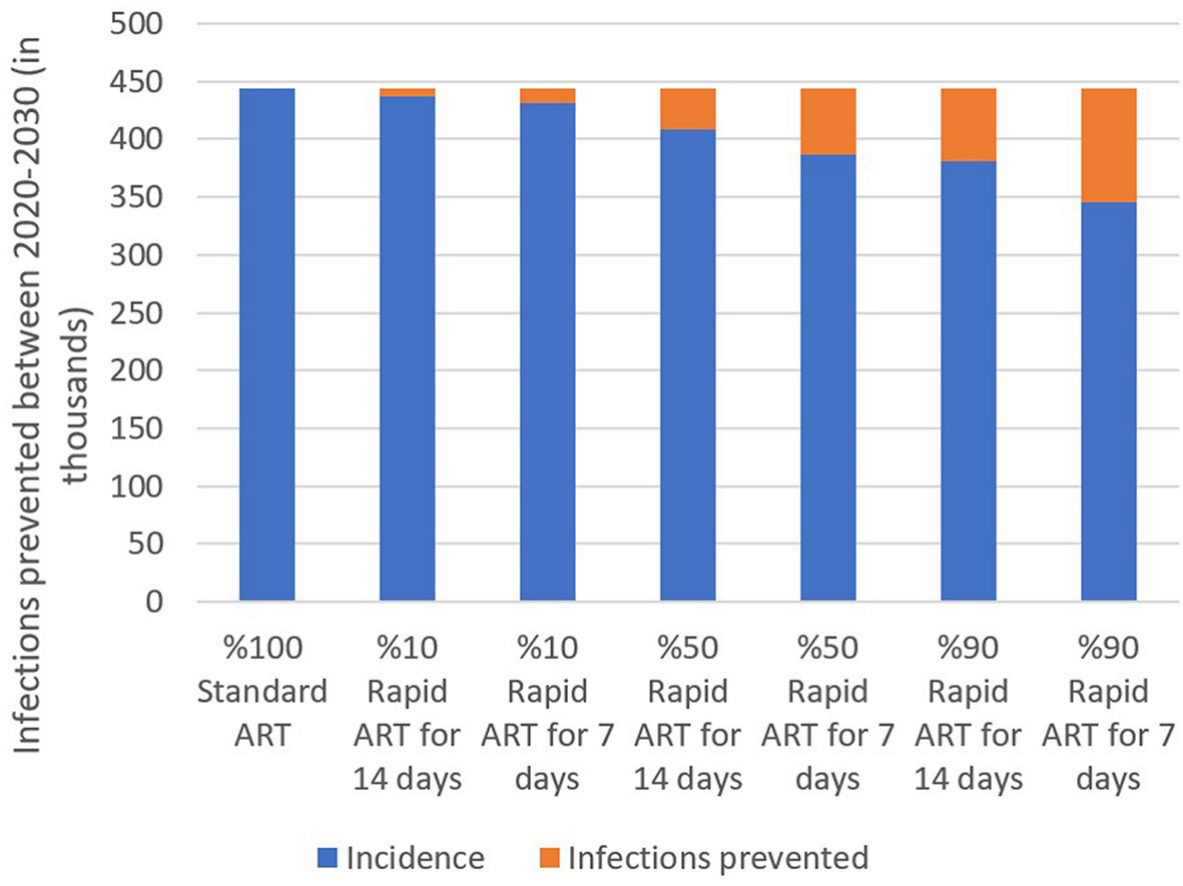
Estimated total incidence and infections prevented under three rapid ART coverage and two rapid ART period scenarios, 2020–2030.

We estimated that additionally 443,682 people would be diagnosed with HIV between 2020 and 2030 if the standard-of-care ART has been continued in Turkey. However, when 10% of diagnosed patients included in the rapid ART program, cumulative HIV incidence would be reduced to 431,858 (3% reduction). With 50% of PLWH included in the rapid ART program, total new cases for a 10-year period would drop to 368,810 (13% reduction). The benefit of the rapid ART program increases to 22% reduction in the cumulative incidence resulting with 345,252 cases when 90% of diagnosed persons receives the rapid ART instead of the standard-of-care (Figure 2).

Furthermore, we investigated the effect of the rapid ART period by analyzing the difference between the rapid ART onset at 7 days after the time of diagnosis and starting this treatment at 14 days after the diagnosis. A 7-day intervention generated less HIV incidence in all coverage scenarios, thereby preventing more infections than the 14-day option (Figure 2). While initiating rapid ART at 7 days leads to 3%, 13%, and 22% reduction in the cumulative incidence as mentioned above, the improvements for 14-days onset were 2%, 8%, and 14%, respectively. The marginal benefit of quicker initiation increases with the coverage level as 90% coverage has shown significant impact of 7-day reduction in the ART initiation. In other words, based on the model results, initiating treatment as early as possible could potentially help in preventing the disease and possibly ending the epidemic.

**Figure 3:**
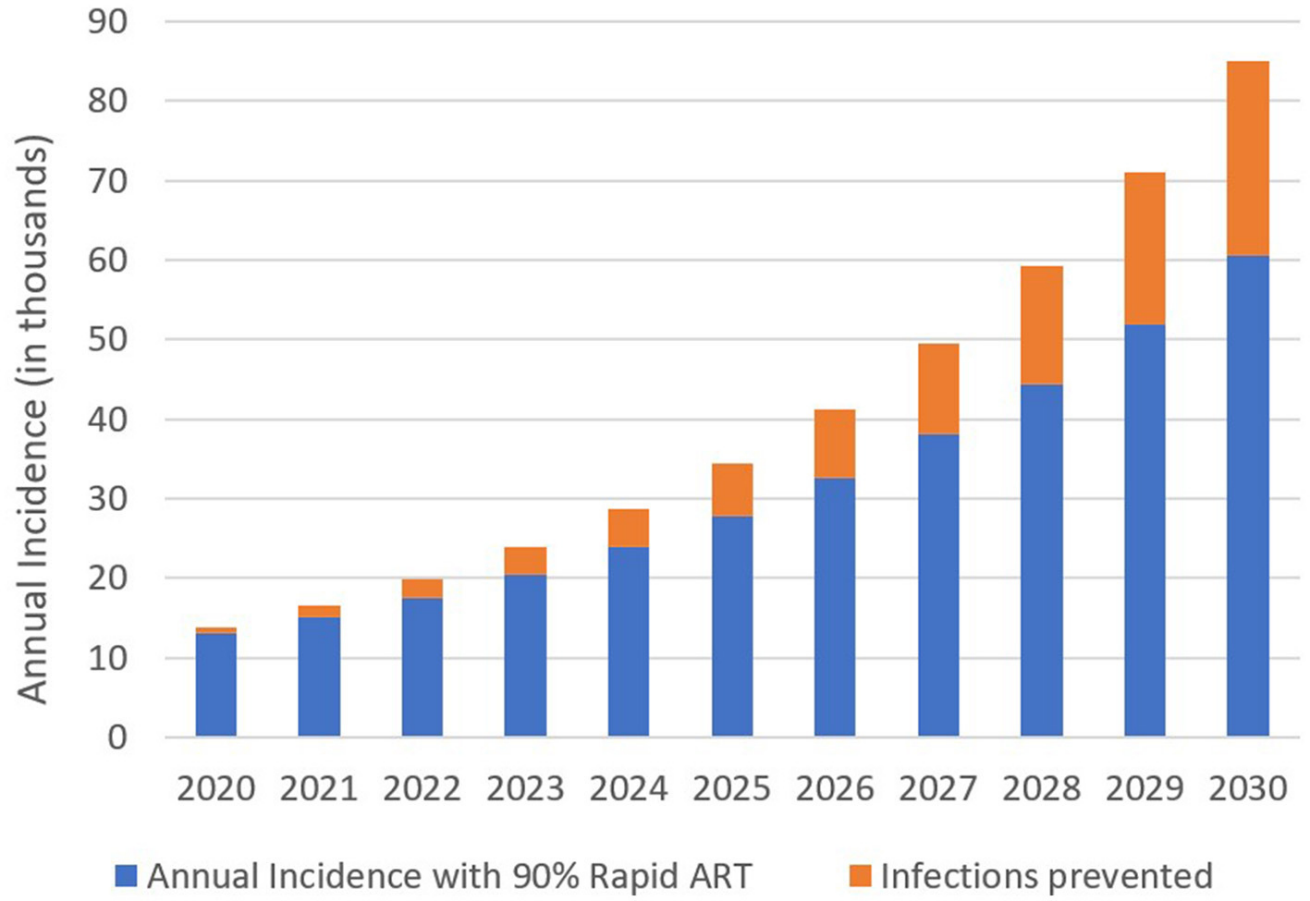
Estimated annual HIV incidence and infection prevented with 90% coverage of the rapid ART intervention, 2020–2030.

We determined annual HIV incidence between 2020 and 2030. Moreover, under the standard-of-care ART, 13,748 new HIV cases have been projected in 2020. HIV incidence increases to 85,130 in 2030 (Figure 3). Introducing the rapid ART program with 90% coverage would reduce HIV incidence to 13,040 and 60,560 for the same years, respectively. We can conclude that the benefits gained from the rapid initiation of ART were increased over the years.

**Figure 4:**
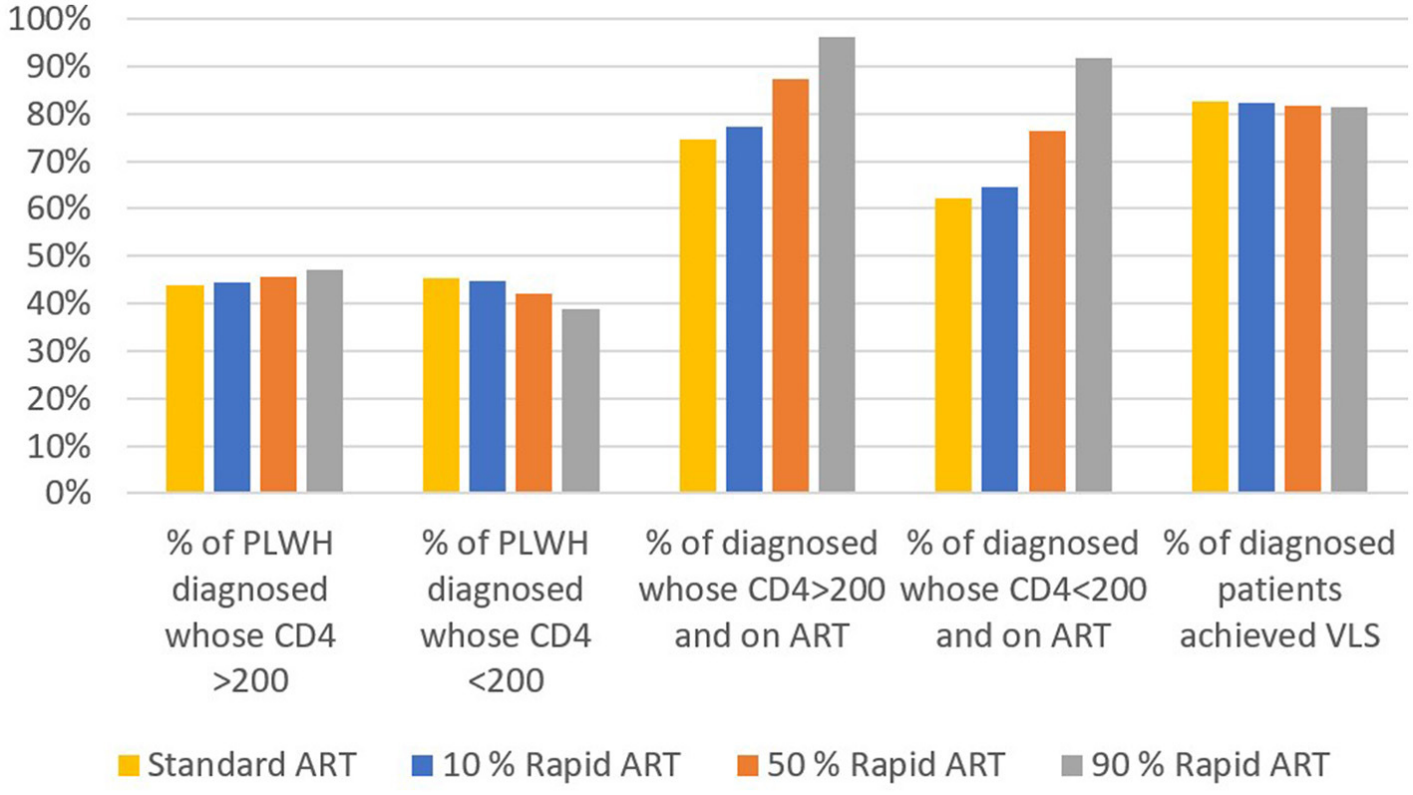
Impact of different rapid ART initiation scenarios on continuum of care.

We calculated the average of 10-year annual percentages of continuum of care for the base case and three rapid ART scenarios (Figure 4). As more patients were offered the rapid ART intervention, the diagnosis rate for PLWH with CD4 count>200 cells/mm^3^ has increased whereas it has decreased for PLWH with CD4 count < 200 cells/mm^3^. On the other hand, the percentage of patients who are on ART has increased significantly for both CD4 levels with the introduction of rapid ART. The final continuum stage, the percentage of patients who achieved VLS among patients on ART, has been remained nearly the same across the different intervention scenarios.

## 4 Discussion

A mathematical model had been adapted to analyze the effectiveness of the rapid ART intervention compared to standard-of-care in Turkey, and the results were evaluated for HIV transmission and progression for a 10-years period. The rapid ART initiation at 7-days and at 14-days were analyzed. Although both interventions reduced the new infectious cases compared to standard care, the initiation of rapid ART at 7 days after the diagnosis had provided better reduction rate in overall HIV incidence. Although we did not analyze the same-day ART initiation, our results suggested that initiating treatment as early as possible is crucial, which is consistent with the previous studies.

In addition, we determined the effect of extending rapid treatment with three coverage levels to a large population. Since there is no current widespread practice of rapid ART programs in Turkey, we defined low, medium, and high coverage with 10%, 50%, and 90% of diagnosed cases who receive the rapid ART, respectively. As expected, the number of prevented infections increases with the increase in the coverage level. Overall, the rapid ART intervention is effective in reducing new HIV infections and it should be considered as a prevention method.

The rapid ART initiation has a positive impact on the continuum of care as the percentage of diagnosed people on ART increases significantly compared to standard care. If the rapid ART reaches 90% coverage, average percentage of PLWH on ART for CD4 count>200 cells/mm^3^ and CD4 count < 200 cells/mm^3^ over a 10-year period was estimated as 96% and 92%, respectively. This implies that, the second part of the 95-95-95 target, i.e., 95% of diagnosed patients will be on ART by 2030 (1), is possible to achieve with the introduction and swift scaling up of the rapid ART initiation. Although the percentage of individuals on ART and achieved VLS among diagnosed did not show any significant changes, as the same efficacy is assumed for both the rapid ART and standard ART in achieving VLS, the same percentages among PLWH improved considerably.

Two sensitivity analyses demonstrated very similar conclusions regarding the most important parameters in the model. The primary drivers of HIV incidence in the model were the force of infection, and the diagnosis rates and interactions among the parameters exist. Force of infection combines the transmission risk between HIV-negative and HIV-positive individuals, mixing among risk populations and HIV prevalence rate by risk populations, and it is the one of main elements that affect the transition between susceptible and infected populations. As a result, it is expected to be one of the influential parameters. The importance of diagnosis rate in this study is probably magnified due to the introduction of rapid ART. With the rapid ART, diagnosed population has been quickly carried to the on ART and VLS compartments where the likelihood of disease transmission is very small. The benefit of compressed timeline accumulates over the time horizon, and changes in the diagnosis rate have a larger effect on the number of new cases.

In Turkey, HIV-positive patients have been eligible to receive ART irrespective of their CD4 level since 2013 guidelines (25); however, we need to take firm actions for ending the HIV due to the evident trend of rising HIV cases in Turkey. To achieve this goal, it is necessary to look for the effective interventions such as rapid ART initiation for patients and, after the careful evaluations including the cost aspect of such interventions, public health policy makers should implement the most beneficial interventions among them. This study provides the evidence on the benefit of the rapid ART intervention on cost and the prevention of new transmissions; therefore, it takes a step toward looking for the effective ways to end the HIV epidemic in Turkey and to achieve the national and international HIV targets.

This study quantifies the effectiveness of rapid ART intervention in Turkey; however, we did not include the behavioral response to the rapid ART as well as implementation barriers. Recent surveys reported that HIV-positive patients in Turkey are mostly in favor of rapid treatment initiation (26). Inclusion of patients in the decision-making process and providing information on treatment pathways to the patients would likely to increase adoption of rapid ART (27). Thus, patients should be well-informed on the process and its results during the implementation phase of the rapid ART programs (3). Barriers to delay the ART onset should be learned and eliminated, as well.

To the best of our knowledge, rapid ART initiation in Turkey has been assessed via a multi-center and retrospective study so far. Considering the lack of standard definition of rapid ART, they categorized the naive PLWH into three groups named as rapid start (RS-ART initiation within the first 24 h after admission to the clinic), early start (ES- ART initiation between the second day and the seventh day after arriving at the clinic), and late start (LS-ART initiation on the eighth day and beyond) groups. In line with other studies, they also found that rapid ART leads to faster viral suppression. Our results are also similar in that there is no significant change in viral suppression rates with rapid ART (18).

Kroon et al. utilized a risk calculator model to estimate the effect of HIV diagnosis during acute infection and immediate initiation of ART combined with behavioral counseling in Thailand. Combining acute HIV infections and early ART with overall behavioral change could potentially result in 89% reduction in the number of onward transmissions across the cohort within the first year of infection while viral load reduction through ART was the most significant contributor to these results. However, the individual effect of early ART is not presented in the study (28). A study conducted by Dimitrov et al. developed a dynamic compartmental model of HIV in Peru and assessed the combination of prevention strategies with early detection and rapid initiation of ART by focusing on the acute HIV phase. After varying different proportions of MSM and transgender women (TW) in the model, the intervention is projected to reduce HIV incidence in 2028 by 24%−60% and new infections over 20 years by 13%−41%. The reported outcomes are common, such as fraction of HIV infections prevented and reduction in HIV incidence, whereas our model differs in assessing the impact of solely rapid initiation of ART (29). Estrada et al. designed a different methodology compared to ours by using a Markov tree to compare rapid ART initiation with current practice of ART in Spain. They had a conservative approach by considering 9 days from HIV diagnosis to rapid ART initiation. They found that rapid ART initiation would prevent about 2% of HIV infections over the 20-year period. Our study results are consistent with this study as they found that initiating ART earlier—at day 7 from diagnosis—averted more HIV cases compared to the initiation at day 9 (30). Krebs et al. investigated several HIV intervention strategies across 6 different US cities employing city-level compartmental models. Rapid ART is defined as same-day ART initiation to newly diagnosed individuals in contrast to our model. Based on the findings of the study, percentage of HIV infections averted over 10-year implementation of rapid ART initiation is estimated between 0.1% and 0.4% for the cities (31). Due to variations in HIV dynamics among different settings, direct comparisons of the magnitude of changes between studies are not feasible. Nevertheless, our findings align with the modeling studies in the literature, suggesting that rapid ART effectively reduces HIV transmission.

There are some limitations to consider when interpreting the results of this study. Since there is no available data that show a change in the parameters over time, we used the same values during the modeling period. Model parameters, such as diagnosis rates and treatment rates, remain constant over the horizon time, which considers the most significant limitation of this study. Another limitation is that we calibrated force of infection parameters due to limited data on sexual and needle sharing behaviors in Turkey. However, we validated the model results with the most recent number of confirmed cases and conducted an extensive sensitivity analysis on this parameter. Apart from these limitations, the analysis of elementary effects showed the parameters that present interactions or nonlinear effects, but it did not reveal which parameters were interacting with each other.

Basic assumption of the compartmental models is homogenous and well-mixed population. Based on this theoretical assumption, all individuals in the population have an equal probability of interacting to each other although there are complex social networks and contact patterns in reality. Our model follows the same assumptions, yet we stratified the model population based on risk groups, such as MSM, PWID, and HET, and defined different infectivity rates for each group to use different infectiousness levels in the population and decrease the limiting effects of the model. We also assumed that the time of the rapid ART initiation and coverage levels are considered the same for all risk groups. We did not consider any behavioral response to rapid ART and assumed that individuals accepted and followed the rapid ART process. Individual-based models, such as network models or agent-based models, could be used for reflecting the heterogeneity among population with the cost of additional computational efforts and increased burden of model inputs.

The strength of our model is that it represents the general dynamics of HIV and enables us to address our research questions with the acceptable number of parameters. Another benefit of our research is that data sets obtained from three largest data source regarded as the most representative data for the country so far are used to create our model. This study facilitates the understanding of the relationships between rapid ART and the number of infections and potentially quantifies the benefit of initiating ART as fast as possible. Furthermore, this study introduces the first comprehensive mathematical model to investigate the effectiveness of rapid ART programs for PLWH in Turkey. The model considers various hypothetical combinations of coverage and initiation times after diagnosis compared to the current standard-of-care ART treatment in the country.

This modeling study provides valuable insights into the significant effect of rapid ART and helps us to understand system behavior under hypothetical conditions. Nevertheless, results should be interpreted with caution due to the aforementioned limitations and methodological simplifications of the model, which do not fully capture the real-world complexities. Moreover, we did not perform any type of economic analysis in this study. The cost aspect of the rapid ART initiation should be investigated with the help of cost-effectiveness analysis before the implementation of such program, particularly for resource-limited settings. For future studies, economic analysis would extend our knowledge of the rapid ART interventions and help to shed a light on the process of selecting the right interventions that achieve the best health outcomes with the least cost for public health decision makers. For future work, the researchers have been encouraged to increase the complexity of the model enhanced by incorporating heterogeneity among the population with different CD4 levels other than 200 cells/mm^3^, age levels or gender when there is available data. Rapid ART applications have recently been started in Turkey, and based on the clinical data that could be collected from these applications, our model could be updated and extended with real-life data and scenarios.

## 5 Conclusion

The rapid ART intervention prevents new HIV cases compared to standard-of-care ART, and the benefit of intervention increases with the coverage and faster initiation. Thus, it can be concluded that the rapid ART initiation could be an effective method to mitigate the increasing trend of HIV cases in Turkey. In other words, the practical implication of the model results is that rapid ART is a promising intervention to replace the existing standard-of-care implementation with the benefits of accelerating entry into medical care, which in turn reduces the HIV transmission in the population. Rapid ART programs demonstrate the potential to provide policy makers with a structural solution for controlling both future direction of HIV and its negative effects on the national healthcare system.

## Data availability statement

The original contributions presented in the study are included in the article/Supplementary material, further inquiries can be directed to the corresponding author.

## Ethics statement

Ethical approval was not required for the study involving humans in accordance with the local legislation and institutional requirements. Written informed consent to participate in this study was not required from the participants or the participants' legal guardians/next of kin in accordance with the national legislation and the institutional requirements.

## Author contributions

EY: Writing – original draft, Conceptualization, Methodology, Funding acquisition, Formal analysis, Software. FC: Writing – review & editing, Conceptualization, Funding acquisition. TS: Conceptualization, Supervision. TO: Conceptualization, Supervision. BO: Conceptualization, Formal analysis, Supervision. HP: Writing – review & editing, Data curation. FY: Writing – review & editing, Data curation. AI: Writing – review & editing, Data curation. OA: Writing – review & editing, Data curation. ST: Writing – review & editing, Data curation. MS: Writing – review & editing, Data curation. ZE: Writing – original draft, Methodology, Formal analysis, Software.
