# Supplementary material for: Rapid initiation of antiretroviral therapy in Turkey: a modeling study
Source: Front Public Health. 2024 Jan 26;12:1224449. doi: 10.3389/fpubh.2024.1224449 (PMC10853345; doi:10.3389/fpubh.2024.1224449)
Supplement: Supplementary file 1 [file Data_Sheet_1.docx]

**Supplementary Material**

**Supplementary Table 1:** Key model parameters

| **Parameters** | **Value** | **Source** | | |
| --- | --- | --- | --- | --- |
| Population parameters |  |  | | |
| Size of the initial population by transmission group |  |  | | |
| HET | 46,863,864 | Calculated from Yıldırım, 2011 ^1^and Data Set | | |
| PWID | 4,239 | Calculated from Data Set | | |
| MSM | 69,897 | Calculated from Data Set | | |
| Percentage of the initial population living with HIV by disease stage, % |  |  | | |
| CD4≥200 | 72.6 | Calculated from the number of diagnosed cases (Ministry of Health) ^2^ | | |
| CD4<200 | 27.4 | Calculated from the number of diagnosed cases (Ministry of Health) ^2^ | | |
| Distribution of the initial population living with HIV across the continuum of care, % |  |  | | |
| Diagnosed among CD4 ≥ 200 PLWH | 47.8 | Calibrated | | |
| Diagnosed among CD4 < 200 PLWH | 47.8 | Calibrated | | |
| on ART among CD4 ≥ 200 PLWDH | 70 | Data Set | | |
| on ART among CD4 < 200 PLWDH | 87 | Data Set | | |
| VLS among on ART PLWDH | 92 | Data Set | | |
| Entry rate (Birth rate) | 4.9x10^-6^ | Turkish Statistical Institute (TÜİK) ^3^ | | |
| The non-HIV related mortality rate | 1.5x10^-5^ | Turkish Statistical Institute (TÜİK) ^3^ | | |
| HIV related mortality rate, % |  |  | | |
| HIV related mortality rate among undiagnosed | 8.54x10^-6^ | Assumed same with diagnosed not on ART | | |
| HIV related mortality rate among diagnosed not on ART | 8.54x10^-6^ | Data Set | | |
| HIV related mortality rate among diagnosed on ART, not VLS | 7.96 x10^-6^ | Data Set | | |
| HIV related mortality rate among VLS | 6.88 x10^-6^ | Data Set | | |
| HIV Progression (rates across horizontal compartments) |  |  | | |
| The natural length of time by disease stage if not on ART | 32 years | CDC Vital Signs^4^ | | |
| Rate of disease stage improvement while undiagnosed & diagnosed not on ART | 0.0066 | Calibrated | | |
| Length of time if on ART | 71 years | CDC Vital Signs^4^ | | |
| Rate of disease stage improvement if on ART not VLS | 0.0012 | Calculated from CDC Vital Signs^4^ | | |
| Length of time if on ART and VLS | 80 years | ^5^ | | |
| Rate of disease stage improvement while VLS | 0.0192 | Calibrated | | |
| Continuum of care progression (rates across vertical compartments) |  |  | | |
| Rate of PLWH that are diagnosed | 0.012 and 0.014 | Calibrated | | |
| Rate of diagnosed PLWH that are on ART | 0.037 and 0.046 | Data Set | | |
| Rate of diagnosed and on ART PLWH that achieve VLS | 0.079 | Data Set | | |
| Rate of dropping out of VLS | 0.002 | Data Set | | |
| Infectivity (Force of infection) | Calibrated |  | | |
| HIV prevalence rate by transmission group |  |  | | |
| HET | 0.01% | Data Set | | |
| PWID | 0.66% | ^6^ | | |
| MSM | 3% | ^7^ | | |
| Reduction in HIV transmission if on ART | 96% | ^8^ | | |
| Reduction in HIV transmission if on VLS | 96% | ^8^ | | |
| Reduction in HIV transmission if the infected person is aware of disease status vs unaware | 53% | ^9^ | | |
| HET: Heterosexuals  PWID: People who inject drugs  MSM: gay, bisexual and other men who have sex with men  PLWH: People Living with HIV  PLWDH: People living with diagnosed HIV  ART: Antiretroviral therapy  VLS: Viral load suppression  The rates were monthly | | |  |  |

**Model formulation**

$\frac{dS_{i}}{dt}=\sigma S_{i}-\frac{S_{i}\left[ \beta_{1}UA_{i}+\beta_{2}DA_{i}+\beta_{3}TA_{i}+\beta_{1}US_{i}+\beta_{2}DS_{i}+\beta_{3}TS_{i} \right]}{N}-\delta S_{i}$ (1)

$\frac{dUA_{i}}{dt}=\frac{S_{i}[\beta_{1}UA_{i}+\beta_{2}DA_{i}+\beta_{3}TA_{i}+\beta_{1}US_{i}+\beta_{2}DS_{i}+\beta_{3}TS]}{N}-k_{1}UA_{i}-pUA_{i}-{rap}_{1}{UA}_{i}-\delta UA_{i}$ (2)

$\frac{dDA_{i}}{dt}=k_{1}UA_{i}-l_{1}DA_{i}-qDA_{i}-\delta DA_{i}$ (3)

$\frac{dTA_{i}}{dt}=l_{1}DA_{i}+{rap}_{1}{UA}_{i}+nVA_{i}-rTA_{i}-mTA_{i}-\delta TA_{i}$ (4)

$\frac{dVA_{i}}{dt}=mTA_{i}+sVS_{i}-nVA_{i}-\delta VA_{i}$ (5)

$\frac{dUS_{i}}{dt}=pUA_{i}-k_{2}US_{i}-\alpha_{1}US_{i}-{rap}_{2}{US}_{i}-\delta US_{i}$ (6)

$\frac{dDS_{i}}{dt}=qDA_{i}+k_{2}US_{i}-l_{2}DS_{i}-\alpha_{2}DS_{i}-\delta DS_{i}$ (7)

$\frac{dTS_{i}}{dt}=rTA_{i}+l_{2}DS_{i}+{rap}_{2}{US}_{i}+nVS_{i}-mTS_{i}-\alpha_{3}TS_{i}-\delta TS_{i}$ (8)

$\frac{dVS_{i}}{dt}=mTS_{i}-nVS_{i}-sVS_{i}-\alpha_{4}VS_{i}-\delta VS_{i}$ (9)

$\frac{dE_{i}}{dt}=\alpha_{1}US_{i}+\alpha_{2}DS_{i}+\alpha_{3}TS_{i}+\alpha_{4}VS_{i}$ (10)

$N=\sum_{i} S_{i}+\sum_{i} UA_{i}+\sum_{i} DA_{i}+\sum_{i} TA_{i}+\sum_{i} VA_{i}+\sum_{i} US_{i}+\sum_{i} DS_{i}+\sum_{i} TS_{i}+\sum_{i} VS_{i}$ (11)

**Supplementary Table 2.** Descriptions of the symbols in the model formula

| **Symbol** | **Description** | **Symbol** | **Description** |
| --- | --- | --- | --- |
| S | Susceptible | $\sigma$ | Birth rate |
| UA | CD4 $\geq$ 200, Undiagnosed | $\delta$ | non-HIV related mortality rate |
| DA | CD4 $\geq$ 200, Diagnosed, not on ART | $\alpha_{1},\alpha_{2},\alpha_{3},\alpha_{4}$ | HIV related mortality rates |
| TA | CD4 $\geq$ 200, on ART, not VLS | $k_{1},k_{2},l_{1},l_{2}$ | Diagnosis rates |
| VA | CD4 $\geq$ 200, on ART, VLS | $n$ | VLS drop-out rate |
| US | CD4 $<$ 200, Undiagnosed | $m$ | VLS achievement rate |
| DS | CD4 $<$ 200, Diagnosed, not on ART | $s$ | Disease stage improvement rate while on VLS |
| TS | CD4 $<$ 200, on ART, not VLS | $p ,q$, $r$ | Disease stage improvement rates |
| VS | CD4 $<$ 200, on ART, VLS | ${rap}_{1}, {rap}_{2}$ | Rapid ART coverage rates |

# **Calibration Process**

Calibration process was applied for unknown parameters in the model consisting the time interval before the rapid initiation program. Force of infection (3x3 matrix), distribution of initial population diagnosed (range 0.25-0.7), rate of disease improvements for three compartments (range 0.02-0.09), and annual diagnosis rates (range 0.01-0.5) were calibrated considering 2010, 2011, 2017 and 2018 target values. These targets were determined by the number of confirmed HIV patients from Turkish Ministry of Health. Since the structure of compartments were designed based on patients’ CD4 level in the model, we used percentages of patients whose CD4 level <200 and CD4 level >200 benefiting from sources such as Ministry of Health and data sets. Thus, we obtained number of diagnosed patients with CD4 >200 and CD4 <200 in each year to apply calibration. Calibration was performed by randomly generating parameter values between their ranges for 10,000 runs. Then, mean squared error (MSE) values were calculated for each parameter set by taking the difference between model incidences and target values. The best parameter set was determined by ranking of parameter sets according to their MSE values. In the second step of calibration process, the best parameter set from first step was used. Then, death rates due to HIV were calibrated by generating random parameter sets for 5,000 run. This time, number of deaths due to HIV for 2017 and 2018 were used as new target values. After that, the model has been run by benefiting from the calibrated parameters over the course of prediction period (2020-2030).

# **Sensitivity Analysis**

The influence of input parameters on the cumulative HIV incidence was analyzed in a one-way sensitivity analysis. We varied all input parameters including the calibrated parameters ±20% unless there was a reported range for the parameter in the literature and reported the results of the analysis with a Tornado graph. Moreover, the nonlinear effects of the parameters due to dynamic nature of the model (i.e. interactions between the parameters) were determined with the elementary effects method, also known as Morris method.

Both sensitivity analysis were applied in the scenario where 50% of diagnosed patients are eligible and offered to be part of rapid ART. It is decided that 23 parameters were included in the second sensitivity analysis which is elementery effects method (Morris Method). Each parameter range was defined between 0 and two times of base value of that parameter. Also, it was assumed that they follow a uniform distribution in their range.

Elementery effects method was performed using the following steps:

- Each range was divided into 20 bins of equal sizes since number of iterations were 20.
- As in Latin Hypercube Sampling (LHS) process, parameter values were randomly generated from each bin. Then, parameter sets was established randomly by taking their values from each bin for each iteration without any repetition.
- Output is defined as total incidence during 2020-2040, and for first parameter set.
- Values of first parameter set was changed by 20% of the range’s width one by one. In other words, only one parameter’s value has changed while others remain the same. When a parameter changed, its new value would be used in the next steps during one iteration. Also, the new parameter value was not allowed to go out of its bounds.
- The outcome was calculated for each parameter set and kept for the next steps.
- This process was applied until all parameters have changed under first iteration.
- The same process until here were repeated for all iterations.
- Outcomes of each iteration were used to calculate the elementery effects of each parameter. When a parameter has been changed, the change in the output was found for that parameter and divided by the rate of change. It means the elementery effect of related parameter. Following formulation was used for the elementery effect of nth parameter:

${EE}_{i,n}= \frac{(Y_{i,n}- Y_{i,n-1})}{\Delta}$

- Mean elementary effects and standard deviation of elementery effects were calculated by taking absolute of elementary effects as follows considering all iterations:

$${\mu_{n}}^{*}= \frac{\sum_{i=1}^{I} \left| {EE}_{i,n} \right|}{I}$$

$$\sigma_{n}= \sqrt{\frac{\sum_{i=1}^{I} {({EE}_{i,n}-\bar{{EE}_{n}})}^{2}}{I}}$$

- The results were interpreted by observing a graph in which x axis represents mean absolute elementery effects (μ*) and y axis shows standard deviation (σ).

# **Sensitivity Analysis Results**

According to 1-way sensitivity analysis, the force of infection had the largest effect on the total HIV incidence, followed by annual diagnosis rates by CD4 count levels (Figure A1). The least effective parameters were HIV-related mortality rates for PLWH on ART and PLWH achieved VLS, and rate of disease stage for PLWH on ART and PLWH achieved VLS.

**Supplementary Figure 1.** Tornado diagram for top 10 effective parameters

Force of infection which quantifies the transmission risk in the model and in Figure A2 were again found to be the most effective parameter with the elementary effects analysis. Similarly, annual diagnosis rates for CD4 count>200 cells/mm^3^ and CD4 count <200 cells/mm^3^, respectively were the other two parameters with a significant impact on the cumulative HIV cases. Considering both standard deviation and mean of absolute elementary effects, these three parameters present interaction effect and non-linearity with others.


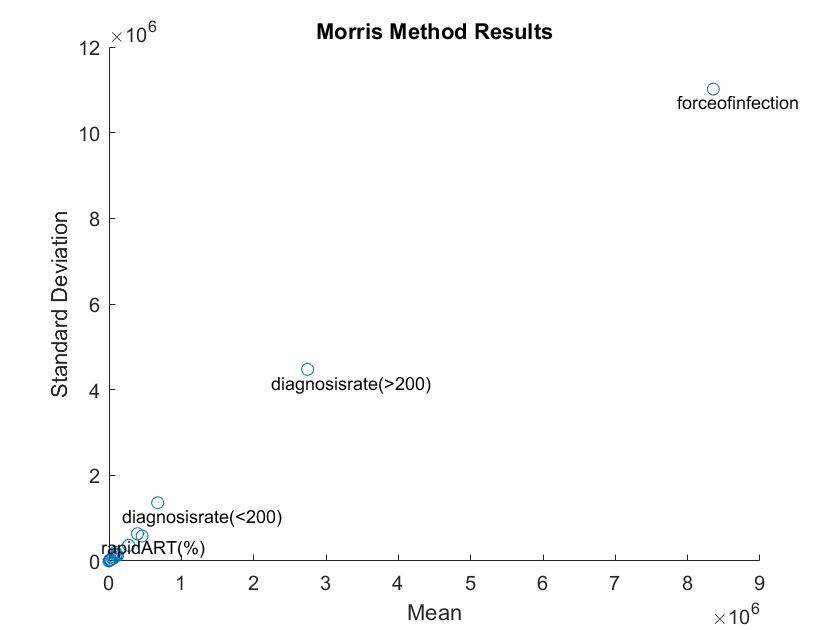


**Supplementary Figure 2.** Results of the elementary effects analysis based on the total HIV incidence between 2020-2030

**References**

1. Yıldırım, K. Avrupa Birliği Ve Türkiye’nin Karşılaştırılmalı Demografik Yapısı. *Sos. Siyaset Konf. Derg.* 31–76 (2011).

2. Turkish Ministry of Health. HIV-AIDS Statistics. (2020).

3. Turkish Statistical Institute. Turkish Statistical Institute (TÜİK). *Birth Statistics, 2019* (2019).

4. Centers for Disease Control and Prevention (CDC) VitalSigns. HIV Care Saves Lives. https://www.cdc.gov/vitalsigns/HIV-AIDS-medical-care/ (2014).

5. May, M. T. *et al.* Impact on life expectancy of HIV-1 positive individuals of CD4R cell count and viral load response to antiretroviral therapy. *AIDS* vol. 28 1193–1202 (2014).

6. İçişleri Bakanlığı, Türkiye Uyuşturucu ve Uyuşturucu Bağımlılığı İzleme Merkezi, A. U. ve U. B. İ. M. *EMCDDA 2013 ULUSAL RAPORU (2012 Verileri) REITOX Ulusal Temas Noktası, Türkiye Yeni Gelişmeler, Trendler, Seçilmiş Konular*. (2013).

7. Marcus, U., Hickson, F., Weatherburn, P. & Schmidt, A. J. Prevalence of HIV among MSM in Europe: Comparison of self-reported diagnoses from a large scale internet survey and existing national estimates. *BMC Public Health* **12**, (2012).

8. Cohen, M. S. *et al.* Prevention of HIV-1 Infection with Early Antiretroviral Therapy. *N. Engl. J. Med.* **365**, 493–505 (2011).

9. Marks, G., Crepaz, N., Senterfitt, J. W. & Janssen, R. S. Meta-Analysis of High-Risk Sexual Behavior in Persons Aware and Unaware They are Infected With HIV in the United States. *JAIDS J. Acquir. Immune Defic. Syndr.* **39**, 446–453 (2005).
